# Association Between Intra-Articular Hyaluronic Acid Injections and Reduction in Analgesic Prescriptions in Knee Osteoarthritis: A Real-World Study from German Outpatient Care

**DOI:** 10.3390/jcm15114153

**Published:** 2026-05-28

**Authors:** Karel Kostev, Marcel Konrad, Christian Tanislav

**Affiliations:** 1 Marburg University, University Hospital, 35043 Marburg, Germany; 2 Epidemiology, IQVIA, 60549 Frankfurt am Main, Germany; 3 Health & Social, FOM University of Applied Sciences for Economics and Management, 60486 Frankfurt am Main, Germany; 4 Department of Geriatrics, Diakonie Hospital Jung Stilling Siegen, 57074 Siegen, Germany; christian.tanislav@diakonie-sw.de

**Keywords:** hyaluronic acid, Recosyn, analgesic, knee osteoarthritis, cohort study

## Abstract

**Background/Objectives:** Intra-articular hyaluronic acid (HA) injections are widely used to treat pain and functional limitations in knee osteoarthritis (KOA). However, evidence on their real-world effectiveness in routine clinical practice and their impact on analgesic medication use remains limited. This study assessed changes in analgesic prescribing following HA injections in a large real-world outpatient population. **Methods:** This retrospective cohort study used data from the IQVIA™ Disease Analyzer database in Germany. Patients with knee osteoarthritis who received a first 20 mg hyaluronic acid injection between 2011 and 2024 and had at least three months of observation before and after treatment were included. Outcomes were (1) the proportion of patients with any analgesic prescription (EPHMRA ATC codes M01A, N02A, N02B) and (2) changes in the number of prescribed analgesic pills within three months before versus after injection. Multivariable logistic regression models evaluated factors associated with post-treatment analgesic prescriptions and reductions in pill counts. **Results:** A total of 4696 patients were included (mean age 64.5 years; 53.9% women), including 524 treated with Recosyn and 4172 treated with other HA products. The proportion of patients with analgesic prescriptions decreased from 28.6% before injection to 26.2% after injection (*p* = 0.004). Among Recosyn-treated patients, the proportion declined from 25.0% to 20.0% (*p* = 0.028). Overall, 70.8% of patients with baseline analgesic use achieved a ≥10% reduction in pill counts and 70.0% achieved a ≥20% reduction. In multivariable analyses, treatment with Recosyn was associated with lower odds of receiving an analgesic prescription after injection (OR 0.67; 95% CI 0.53–0.85) and higher odds of achieving a ≥10% reduction in analgesic pill count (OR 1.64; 95% CI 1.05–2.58). **Conclusions:** In routine outpatient practice, HA injections were accompanied by modest reductions in analgesic prescribing among patients with KOA. Numerically greater reductions were observed among patients treated with Recosyn compared with other HA products; however, these findings should be interpreted with caution given the observational design, potential residual confounding, and the absence of a non-treated comparator group. These results should be considered hypothesis-generating.

## 1. Introduction

Knee osteoarthritis (KOA) is a chronic and progressive joint disease characterized by cartilage degeneration, subchondral bone remodeling, synovial inflammation, pain, and functional limitation. It represents one of the most common causes of disability worldwide, affecting an estimated 595 million people in 2020, with projections indicating further substantial increases due to population aging and growth [1]. Major risk factors include older age, female sex, obesity, prior joint injury, and biomechanical or occupational stress [2].

The burden of KOA is considerable at both individual and societal levels. Patients frequently experience chronic pain, reduced mobility, and impaired quality of life, which can limit activities of daily living and work participation. From a health-system perspective, KOA is associated with increased healthcare utilization, including outpatient visits, imaging procedures, pharmacological therapy, rehabilitation, and surgical interventions. Because definitive disease-modifying treatments remain limited and joint replacement is typically reserved for advanced disease, effective symptom management strategies are essential to reduce disability and healthcare demand [1,3].

Management of KOA generally follows a stepwise approach combining non-pharmacological and pharmacological interventions. Non-steroidal anti-inflammatory drugs (NSAIDs) and other analgesics are commonly used for pain relief [4,5]. Intra-articular corticosteroid injections may provide short-term symptomatic benefit in selected patients [6]. For patients with severe disease or persistent symptoms despite conservative therapy, surgical interventions such as total knee arthroplasty remain effective but resource-intensive treatment options [2].

Intra-articular hyaluronic acid (HA) injections are widely used as a treatment option for KOA pain and functional limitation. HA is a natural component of synovial fluid and cartilage extracellular matrix, contributing to joint lubrication, viscoelasticity, and shock absorption, and may also exert anti-inflammatory and chondroprotective effects [7]. Numerous randomized trials and meta-analyses have evaluated the efficacy of HA injections, generally reporting modest but statistically significant improvements in pain and function compared with placebo or baseline, although treatment effects vary substantially across studies [8,9,10,11,12]. Evidence also suggests that formulation characteristics such as molecular weight or injection protocols may influence treatment outcomes [7,13]. Clinical studies also explore alternative injection regimens such as single-injection highly concentrated HA preparations [14]. Other intra-articular approaches, including platelet-rich plasma, have been investigated and may demonstrate different comparative efficacy profiles in some analyses [15].

Despite the growing number of randomized clinical trials and meta-analyses, evidence from routine clinical practice remains limited. Clinical trials are typically conducted under controlled conditions with strict inclusion and exclusion criteria—such as narrow age limits, exclusion of patients with relevant comorbidities, and standardised injection protocols—which may not fully reflect the heterogeneity of patients encountered in everyday care, including variations in disease severity, comorbidities, concomitant treatments, and treatment pathways. As a result, trial populations often differ substantially from the broader population seen in routine practice, where patients more frequently present with multimorbidity and polypharmacy. Consequently, the external validity of trial findings may be limited when applied to routine clinical settings, and effect estimates from trials may over- or underestimate the benefit–risk profile expected in real-world practice. Real-world studies are therefore important to evaluate treatment outcomes in broader patient populations and to assess endpoints relevant to daily clinical practice.

The primary objective of this study was to evaluate whether initiation of intra-articular 20 mg hyaluronic acid (HA) injections is associated with a reduction in analgesic medication use within three months of treatment in patients with knee osteoarthritis (KOA) treated in routine German outpatient practice. As a secondary objective, we examined whether the magnitude of this association differed between patients treated with the Recordati HA product Recosyn and those receiving other commercially available HA preparations.

## 2. Methods

### 2.1. Data Source

The data for this study were obtained from the Disease Analyzer database, which contains anonymized longitudinal patient information from general practitioners and specialists in Germany. The database includes routinely collected data on diagnoses, prescriptions, and basic demographic characteristics documented in physicians’ electronic medical records. Patient data are transmitted from participating practices to IQVIA on a monthly basis using standardized interfaces and are anonymized before inclusion in the database to ensure compliance with data protection regulations. The Disease Analyzer database has been widely used for epidemiological and pharmacoepidemiological research and has been shown to be broadly representative of the German outpatient population with respect to patient characteristics and disease distribution [16].

### 2.2. Study Design and Outcomes

This retrospective cohort study represents a real-world evidence (RWE) analysis based on routine outpatient care data. Inclusion criteria were: (1) a documented ICD-10 diagnosis of knee osteoarthritis (M17) recorded prior to the index date; (2) receipt of a first-ever intra-articular 20 mg hyaluronic acid injection between January 2011 and December 2024 (index date); (3) continuous database enrolment for at least three months before the index date; and (4) at least three months of follow-up after the index date. Exclusion criteria were: absence of a recorded M17 diagnosis, and insufficient pre- or post-index observation time. Radiographic grading of osteoarthritis severity (e.g., Kellgren–Lawrence grade) is not captured in the Disease Analyzer database; accordingly, the study population likely includes patients with mild-to-severe disease.

For these patients, two outcomes were evaluated:

1. Prescription of pain medication (EPHMRA ATC codes: M01A, N02A, N02B), assessed as a binary outcome (yes/no) within three months after the index date compared with the three months prior.
2. Total number of pain medication pills per patient within three months after the index date compared with the three months prior.

Total analgesic pill counts were derived from prescribed pack sizes across different analgesic classes, including NSAIDs, non-opioid analgesics, and opioids. Because these drug classes differ substantially in pharmacological potency and dosing regimens. Because these drug classes differ substantially in pharmacological potency and dosing regimens, pill counts were not interpreted as equivalent measures of analgesic exposure. Instead, they were used as a pragmatic indicator of overall prescribing intensity and frequency of analgesic treatment in routine outpatient care. Class-specific analyses were not performed because analgesic switching between classes after HA injection was common in this real-world population. Separate analyses for individual analgesic classes could therefore produce misleading interpretations, as reductions in one class may reflect substitution by another class rather than a true reduction in overall analgesic treatment burden. The selected approach was intended to better capture overall changes in pharmacological pain management irrespective of the specific analgesic class prescribed.

Analyses were conducted for all patients receiving HA injection and were further stratified by HA product type (Recosyn vs. other products), sex (women vs. men), age group (<60, 60–69, ≥70 years). The chosen age stratification (<60, 60–69, ≥70 years) was intended to reflect clinically meaningful stages of osteoarthritis management while ensuring sufficient sample sizes within each subgroup for statistical analysis. In the present study population, this categorization allowed a balanced distribution of patients across age groups and facilitated comparisons between younger patients, those approaching older age, and patients in clearly older age groups. Subgroup analyses were considered exploratory and descriptive. No formal adjustment for multiple comparisons was applied; therefore, subgroup findings should be interpreted with caution due to the potential for type I error.

Given the observational pre–post design without an untreated comparator group, the analysis is intended to assess associations between HA injection and changes in analgesic prescribing rather than to establish causal effects.

### 2.3. Statistical Analysis

In the first step, the proportion of patients with any analgesic prescription within three months prior to versus three months after the HA injection was compared using the McNemar test.

To assess whether pill consumption changed between the pre- and post-injection periods, paired differences in the total number of prescribed analgesic pills (post minus pre) were evaluated using the Sign test. This non-parametric test assesses whether the direction of change differs systematically between the two periods. Analyses were performed in the overall population and stratified by sex, age group (<60, 60–69, ≥70 years), and HA product (Recosyn vs. other HA products).

Among patients with at least one analgesic prescription within three months prior to injection, we additionally evaluated the proportion of patients with any reduction in analgesic pill count as well as reductions of at least 10% and at least 20%. These thresholds were used to distinguish moderate and more pronounced reductions in analgesic prescribing. Differences between patients treated with Recosyn and those treated with other HA products were compared using the chi-square test.

Multivariable logistic regression models were applied to examine associations between patient characteristics and the likelihood of receiving any analgesic prescription within three months after HA injection. Models adjusted for prior analgesic prescriptions, age, sex, health insurance status (statutory vs. private), physician specialty (orthopaedist vs. general practitioner), and treatment with Recosyn.

Additional logistic regression models were used to evaluate factors associated with reductions in analgesic pill count of at least 10% and at least 20%, adjusting for baseline pill count, age, sex, health insurance status, physician specialty, and treatment with Recosyn.

A two-sided *p*-value < 0.05 was considered statistically significant. All statistical analyses were performed using SAS version 9.4 (SAS Institute, Cary, NC, USA).

## 3. Results

### 3.1. Baseline Characteristics

A total of 13,003 patients received their first HA injection (20 mg) between 2010 and 2024 (index date). Of these, 5951 patients had at least three months of observation prior to the index date and at least three months of follow-up after the index date. Among them, 4696 patients had a diagnosis of gonarthrosis (osteoarthritis of the knee; ICD-10: M17) and were included in the final analysis. A total of 524 patients received Recosyn, while 4172 patients were treated with other HA products. The number of prescribed injections was the same in both groups (median: 5.0; IQR: 0).

Among the 4696 patients treated with HA injections, the mean age was 64.5 years (SD 13.5). Patients receiving Recosyn were slightly older on average than those treated with other HA products. Overall, the age distribution indicated that a substantial proportion of patients were older adults, with a notable representation of individuals aged 70 years or above, particularly in the Recosyn group compared with those receiving other HA formulations. Women accounted for 53.9% of all patients, with a higher proportion observed among those treated with Recosyn than among those receiving other HA products. In terms of insurance status, patients treated with Recosyn were more frequently covered by statutory health insurance, whereas those receiving other HA products were more commonly privately insured. Most patients were treated by orthopaedists, while a smaller proportion received care from general practitioners, with only minor differences between treatment groups (Table 1).

### 3.2. Prescription of Pain Medication Assessed as a Binary Outcome

**Figure 1:**
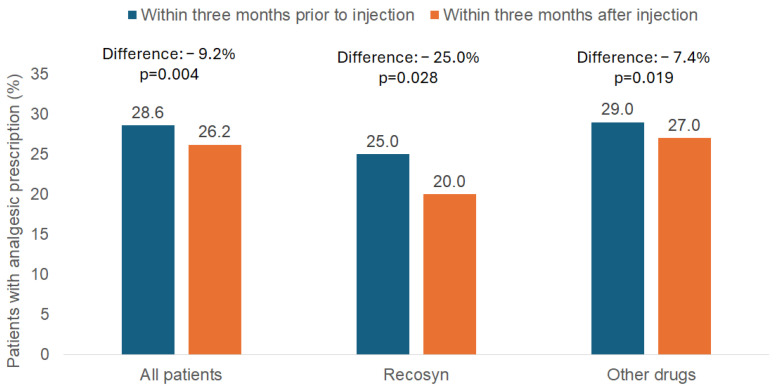
Proportion of patients with at least one analgesic prescription within three months before and after HA injection (overall population). Differences were assessed using the McNemar test.

Figure 1 displays the proportion of patients who received at least one analgesic prescription during the three months prior to the first HA injection and during the three months following the injection. In the total population, the proportion decreased from 28.6% before injection to 26.2% after injection, representing a relative reduction of 9.2% and a McNemar *p*-value of 0.004.

Among patients treated with Recosyn, the proportion of individuals with analgesic prescriptions declined from 25.0% prior to injection to 20.0% after injection, corresponding to a relative reduction of 25.0% and a *p*-value of 0.028. Among patients treated with other HA drugs, the proportion changed from 29.0% before to 27.0% after injection, a relative reduction of 7.4% with a *p*-value of 0.019.

**Figure 2:**
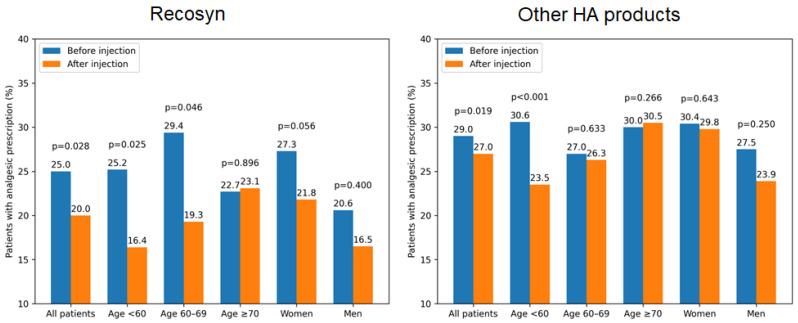
Proportion of patients with analgesic prescriptions before and after HA injection, stratified by age and sex. Differences were assessed using McNemar tests. Subgroup analyses are descriptive.

Figure 2 also illustrates the age-specific patterns. In patients younger than 60 years, the proportion of analgesic users decreased from 30.0% to 22.8%, a relative reduction of 24.0% with a *p*-value < 0.001. For patients aged 60–69 years, the proportions were 27.3% before and 25.6% after injection, a relative reduction of 6.2% and a *p*-value of 0.288. In patients aged 70 years or older, the proportion rose slightly from 28.2% prior to 29.6% after injection, resulting in a difference of +5.0% and a *p*-value of 0.274. When stratified by sex, women showed proportions of 30.0% before and 28.7% after the injection, corresponding to a difference of −4.3% and a *p*-value of 0.264. Men had proportions of 26.9% before and 23.3% after injection, a difference of −13.4% and a *p*-value of 0.262.

Among Recosyn-treated patients, the most frequently prescribed analgesics before and after injection were diclofenac, metamizole sodium, ibuprofen, tilidine + naloxone, and etoricoxib. The number of patients receiving analgesic prescriptions decreased from 163 before treatment to 125 after treatment. Among patients receiving other HA products, the most commonly prescribed analgesics were ibuprofen, diclofenac, metamizole sodium, etoricoxib, and tilidine + naloxone. The number of patients with analgesic prescriptions decreased from 1529 before treatment to 1453 after treatment.

### 3.3. Pill Count Change

The distribution of pill counts was highly skewed, with a large proportion of patients having no prescriptions during the observation periods, resulting in median values of zero in several groups. Therefore, median values should be interpreted with caution and primarily reflect the high frequency of zero values rather than the full distribution of prescribing patterns. Given this distributional feature, pill count data should not be overinterpreted as a clinically meaningful outcome in isolation; the proportion-based endpoint (any analgesic prescription before vs. after injection) and the ≥10%/≥20% reduction analyses among patients with baseline prescriptions are considered the more informative primary endpoints in this dataset.

In the overall population, the median pill count was 0 (IQR 0–20) before injection and 0 (IQR 0–7) after injection. The sign test indicated a statistically significant change in pill counts between the two periods (test statistic = −44, *p* = 0.044). In the Recosyn subgroup, both pre- and post-injection medians were 0 (IQR 0–1 and 0–0, respectively), with a median difference of 0 (IQR 0–0), a sign test statistic of −16, and a *p*-value of 0.018. Among patients receiving other HA products, medians remained 0 (IQR 0–20 both before and after), with a difference of 0 (IQR 0–0), a sign test statistic of −28, and a *p*-value of 0.184.

**Figure 3:**
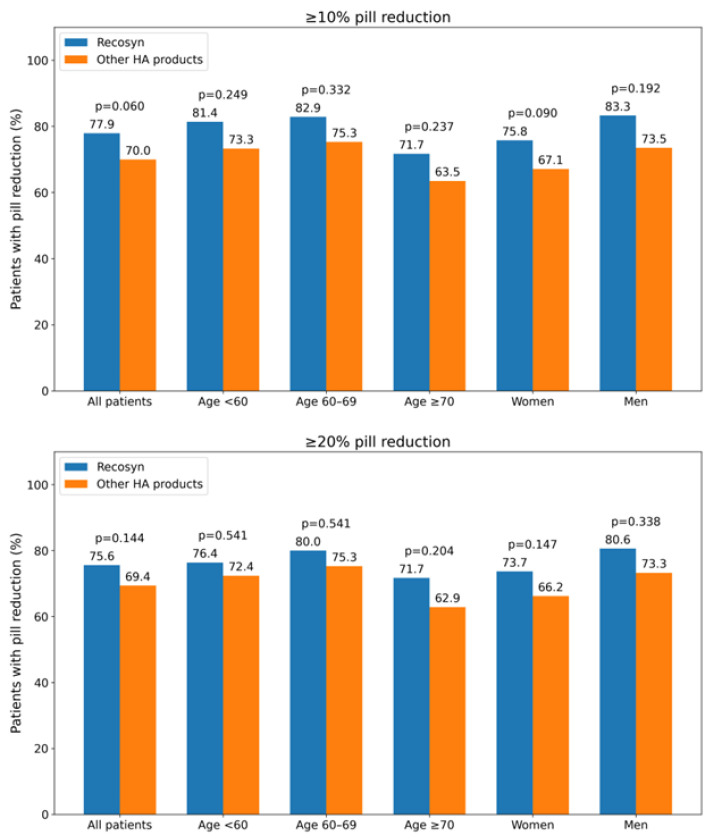
Proportion of patients achieving ≥10% and ≥20% reductions in analgesic pill counts, stratified by age group and HA product. Differences between groups were assessed using chi-square tests. Analyses are exploratory.

For a ≥10% pill count reduction, 70.8% of all patients with baseline use achieved this level of reduction. Among Recosyn patients, 77.9% experienced a ≥10% reduction compared with 70.0% among patients treated with other HA products (*p* = 0.060). For ≥20% pill reduction, 70.0% of all patients reached this threshold. The proportion was 75.6% among Recosyn users and 69.4% among other drug users (*p* = 0.144) (Figure 3).

### 3.4. Regression Analysis

Having an analgesic prescription within the three months prior to injection was associated with higher odds of receiving an analgesic prescription after injection (OR 2.86; 95% CI 2.48–3.30). Older age (≥70 years) was associated with increased odds of receiving an analgesic prescription after injection compared with younger age (<60 years) (OR 1.45; 95% CI 1.23–1.71). Male sex was associated with lower odds of receiving an analgesic prescription after injection (OR 0.75; 95% CI 0.65–0.86). Treatment by an orthopaedist was associated with lower odds of receiving an analgesic prescription after injection compared with treatment by a general practitioner (OR 0.41; 95% CI 0.34–0.49). Use of Recosyn was associated with lower odds of receiving an analgesic prescription after injection compared with other hyaluronic acid products (OR 0.67; 95% CI 0.53–0.85) (Table 2).

A higher baseline analgesic pill amount (per 10 pills) was associated with lower odds of achieving at least a 10% reduction (OR 0.98; 95% CI 0.96–1.00). Older age (≥70 years) was associated with lower odds of achieving at least a 10% reduction compared with younger age (<60 years) (OR 0.67; 95% CI 0.55–0.88). Male sex was associated with higher odds of achieving at least a 10% reduction (OR 1.37; 95% CI 1.07–1.75). Treatment by an orthopaedist was associated with higher odds of achieving at least a 10% reduction compared with treatment by a general practitioner (OR 2.28; 95% CI 1.71–3.04). Use of Recosyn was associated with higher odds of achieving at least a 10% reduction compared with other hyaluronic acid products (OR 1.64; 95% CI 1.05–2.58). Age 60–69 years was not significantly associated with achieving a 10% reduction compared with age <60 years (OR 1.14; 95% CI 0.82–1.59).

A higher baseline analgesic pill amount (per 10 pills) was also associated with lower odds of achieving at least a 20% reduction (OR 0.97; 95% CI 0.96–0.99). Older age (≥70 years) was associated with lower odds of achieving at least a 20% reduction compared with younger age (<60 years) (OR 0.70; 95% CI 0.53–0.92). Male sex was associated with higher odds of achieving at least a 20% reduction (OR 1.41; 95% CI 1.10–1.81). Treatment by an orthopaedist was associated with higher odds of achieving at least a 20% reduction compared with treatment by a general practitioner (OR 2.26; 95% CI 1.70–3.00). Use of Recosyn was associated with higher odds of achieving at least a 20% reduction compared with other hyaluronic acid products, although this association did not reach statistical significance (OR 1.51; 95% CI 0.97–2.34). Age 60–69 years was not significantly associated with achieving a 20% reduction compared with age <60 years (OR 1.20; 95% CI 0.86–1.67) (Table 3).

## 4. Discussion

This real-world study examined changes in prescribed analgesic use before and after intra-articular hyaluronic acid (HA) injections in nearly 4700 patients with knee osteoarthritis treated in German outpatient practices. Overall, HA injections were associated with modest but significant reductions in both the proportion of patients receiving analgesic prescriptions and the volume of prescribed analgesic pills during the three months following treatment. These reductions were more pronounced among patients treated with Recosyn than among those receiving other HA preparations. Treatment with Recosyn was independently associated with lower odds of post-injection analgesic prescriptions and with higher odds of achieving a clinically relevant reduction in analgesic pill counts in multivariable analyses. The more pronounced reductions in analgesic use observed among patients treated with Recosyn should be interpreted with caution. The non-randomized allocation of HA products and the baseline differences between treatment groups suggest potential confounding by indication and channeling bias. Although multivariable adjustment was performed for several key variables, residual confounding due to unmeasured factors—such as disease severity, symptom burden, or physician treatment preferences—cannot be excluded. These factors may partly explain the observed differences between products. Differences in baseline characteristics between treatment groups should therefore be considered when interpreting these findings. Nevertheless, the observed association between Recosyn treatment and reduced analgesic use remained consistent across multiple exploratory analyses after adjustment for measured covariates, although residual confounding cannot be excluded. However, these findings should be interpreted as hypothesis-generating.

The direction of these findings is consistent with evidence from randomized trials and meta-analyses showing that viscosupplementation can provide symptomatic improvement in knee osteoarthritis, although the magnitude of benefit varies across studies. A systematic review and meta-analysis by Pereira et al. (2022) reported statistically significant improvements in pain and function with viscosupplementation compared with placebo; however, the authors noted that the absolute effect sizes—expressed as standardised mean differences—were modest and, in several analyses, below the threshold commonly regarded as the minimal clinically important difference [17]. This means that, on average, improvements reached statistical significance but the practical magnitude of benefit for individual patients may be limited; nevertheless, a subset of patients may respond more substantially, and the identification of predictors of treatment response remains an active area of research. Similarly, a meta-analysis of randomized trials involving more than 8000 patients confirmed the favorable safety profile of intra-articular HA injections [18]. Comparative analyses of pharmacologic treatments for knee osteoarthritis have also shown that HA injections are associated with measurable reductions in pain compared with placebo [19]. More recently, model-based analyses have highlighted that treatment response may depend on patient characteristics, disease severity, and study design, which may partly explain the heterogeneous findings reported across studies [11]. The present results, derived from routine outpatient practice and using analgesic prescriptions as a proxy outcome, align with this broader evidence base. This real-world setting may allow the exploration of formulation- or practice-related differences beyond those typically captured in randomized trials.

The observed reductions in analgesic prescribing should have clinical relevance, particularly in older patients with knee osteoarthritis who frequently present with multimorbidity and polypharmacy. Long-term use of analgesic medications, especially non-steroidal anti-inflammatory drugs and opioid agents, is associated with increased risks of adverse drug reactions and drug–drug interactions. In particular, NSAIDs may increase the risk of cardiovascular events and gastrointestinal complications, while opioid therapy can be associated with central nervous system effects and dependency [20,21]. Consequently, strategies that reduce the need for analgesic therapy will be clinically beneficial, even when absolute reductions in prescription numbers appear modest. The absolute reductions in analgesic prescription rates observed in this study—approximately 2.4 percentage points in the overall population and 5 percentage points in the Recosyn subgroup—should be considered in the context of the resource implications of intra-articular HA therapy. A standard course typically involves multiple injections (median five injections in this cohort), each requiring an outpatient visit and carrying a small but non-zero risk of procedure-related adverse events, including post-injection flare and infection. Common analgesics such as paracetamol or low-dose ibuprofen are substantially less expensive and are associated with fewer procedural risks when used short-term in selected patients. Formal cost-effectiveness analyses were beyond the scope of the present study; however, the modest absolute effect sizes observed here would likely require demonstration of additional benefits—such as improvements in patient-reported pain scores, functional outcomes, or quality of life—to justify viscosupplementation on cost-effectiveness grounds alone. Future studies combining prescription data with patient-reported outcomes and health economic analyses would be valuable in this regard.

The more pronounced reductions in analgesic use observed among patients treated with Recosyn should be interpreted with caution. Although all treatments involved 20 mg hyaluronic acid injections, the database does not include detailed information on formulation characteristics such as molecular weight, cross-linking, or viscoelastic properties, nor on injection protocols.

Product-focused reviews indicate that molecular weight, concentration, and structural properties of HA preparations influence viscoelastic behavior and intra-articular residence time [22,23]. In particular, higher molecular weight formulations may enhance mechanical lubrication and shock absorption within the joint, potentially contributing to symptomatic improvement [24]. Evidence from studies in hip osteoarthritis also suggests that molecular weight can affect both the efficacy and safety profile of HA viscosupplementation [25]. Taken together, these mechanistic considerations may provide a possible explanation for the observed differences between HA products; however, these hypotheses cannot be directly evaluated within the present dataset and should therefore be interpreted as speculative.

Several limitations should be considered when interpreting the present findings. First, the database contains information on prescribed medications but does not capture over-the-counter (OTC) analgesic use. Second, analgesic prescriptions were used as a clinically relevant endpoint reflecting physicians’ treatment decisions in response to patients’ pain symptoms. However, because patient-reported pain scores are typically unavailable in administrative databases, prescription data represent an objective proxy for pain management in routine clinical practice [26,27]. Third, the analysis captured only prescription analgesics classified under EPHMRA ATC codes M01A, N02A, and N02B. Topical NSAID formulations (e.g., diclofenac gel) that are widely available without prescription in Germany are not recorded in the Disease Analyzer database. Similarly, prescription topical analgesic preparations dispensed under other ATC codes may not have been captured. As topical analgesics are frequently used in knee osteoarthritis, particularly in older patients, the present analyses may underestimate total analgesic use and may not fully reflect the clinical pain management burden of the study population. More broadly, the analyses reflect only prescription-based analgesic therapy and may underestimate total analgesic consumption. Fourth, the database relies on routine clinical documentation and ICD-10 coding, which does not allow detailed assessment of clinical parameters such as pain severity, functional status, or radiographic disease stage. Patient-reported pain scores (e.g., VAS, NRS), functional assessments (e.g., WOMAC, KOOS), and measures of walking ability or quality of life are not available. Consequently, it is not possible to determine whether reductions in analgesic prescribing translate into clinically meaningful improvements in pain or physical function, as prescribing decisions are influenced by multiple factors including physician preference, patient adherence, contraindications, and over-the-counter substitution. The present findings should therefore be interpreted as real-world pharmacoepidemiological evidence on prescribing patterns rather than as a demonstration of clinical efficacy. Imaging findings, lifestyle factors, and socioeconomic variables are also not available. Furthermore, the ICD-10 code M17 encompasses all clinical grades of knee osteoarthritis, and radiographic staging (e.g., Kellgren–Lawrence grade) is not available in the database; the study population therefore likely includes patients with mild-to-severe disease. Fifth, the database captures outpatient care and therefore may not include events managed exclusively in hospital settings. Sixth, given the number of subgroup and secondary analyses performed, these findings may be subject to multiplicity and should be interpreted as exploratory. Seventh, although the number of injections was comparable between treatment groups, suggesting similar treatment intensity, the present analysis did not incorporate detailed product-level characteristics—such as molecular weight or structural properties—into the statistical models, limiting the ability to attribute observed differences to specific formulation features. Eighth, the analysis of analgesic pill counts is limited by the highly skewed distribution with a substantial proportion of zero values, which reduces the informativeness of summary measures such as medians. To address this, additional endpoints such as the presence of any prescription and relative reductions in pill counts were included to better capture changes in prescribing behavior. These complementary measures provide a more comprehensive assessment of treatment-associated changes than pill counts alone in real-world datasets with sparse prescribing.

Importantly, the pre–post observational design without an untreated comparator group substantially limits causal inference. Observed changes in analgesic prescribing may reflect regression to the mean, secular trends, or natural fluctuations in symptom severity rather than a direct effect of HA injections alone. Nevertheless, the consistency of findings across multiple outcomes and their persistence after adjustment for key covariates suggest that the observed associations are robust, although they should be interpreted as hypothesis-generating.

Finally, the comparator category included multiple HA formulations with potentially different physicochemical characteristics and clinical use patterns. Consequently, the comparison between Recosyn and other HA products should not be interpreted as a definitive head-to-head comparative effectiveness analysis but rather as an exploratory comparison within routine clinical practice.

Despite these limitations, the present study provides real-world evidence on treatment outcomes following HA injections in a large outpatient population. The findings showed that HA injections are associated with reductions in prescribed analgesic use in routine clinical practice and that differences between HA products may contribute to heterogeneous real-world outcomes; however, these observations require confirmation in studies specifically designed for comparative effectiveness analyses.

## 5. Conclusions

In conclusion, intra-articular hyaluronic acid injections were associated with modest reductions in analgesic use in routine clinical practice, with numerically greater reductions observed among patients treated with Recosyn, although these differences should be interpreted cautiously given the observational design and limited product-level information.

## Figures and Tables

**Table 1:** Baseline characteristics of the study population (n = 4696), including patients treated with Recosyn (n = 524) and other HA products (n = 4172). Values are proportions (%) unless otherwise indicated. *p*-values refer to comparisons between treatment groups using chi-square tests for categorical variables and t-tests for continuous variables.

Variable	Proportion Among HA Patients (n = 4696)	Proportion Among Individuals with Recosyn (n = 524)	Proportion Among Individuals with Other Drugs (n = 4172)	*p*-Value
Age (Mean, SD)	64.5 (13.5)	65.7 (13.1)	64.3 (13.6)	0.030
Age < 60 years	1576 (33.6)	171 (32.6)	1407 (33.7)	0.001
Age 60–69 years	1258 (26.8)	119 (22.7)	1139 (27.3)	
Age ≥ 70 years	1860 (39.6)	234 (44.7)	1626 (39.0)	
Women	2530 (53.9)	348 (66.4)	2182 (52.3)	<0.001
Men	2166 (46.1)	176 (33.6)	1990 (47.7)	
Statutory health insurance coverage	2267 (48.3)	441 (84.2)	1826 (43.8)	<0.001
Private health insurance coverage	2429 (51.7)	83 (15.8)	2346 (56.2)	
Treated by general practitioner	680 (14.5)	61 (11.6)	610 (14.8)	0.050
Treated by orthopedist	4016 (85.5)	463 (88.4)	3553 (85.2)	

Proportions of patients in % given, unless otherwise indicated. SD: standard deviation.

**Table 2:** Association between baseline characteristics, Recosyn therapy and any analgesic prescription post-index (multivariable logistic regression model).

Variable	Odds Ratio (95% CI)	*p*-Value
Analgesic prescription within three months prior to injection	2.86 (2.48–3.30)	<0.001
Age < 60 years	Reference	
Age 60–69 years	1.18 (0.99–1.43)	0.065
Age ≥ 70 years	1.45 (1.23–1.71)	<0.001
Women	Reference	
Men	0.75 (0.65–0.86)	<0.001
Statutory health insurance coverage	Reference	
Private health insurance coverage	0.99 (0.86–1.14)	0.882
Treated by general practitioner	Reference	
Treated by orthopedist	0.41 (0.34–0.49)	<0.001
Recosyn	0.67 (0.53–0.85)	0.001
Other drugs	Reference	

**Table 3:** Association between baseline characteristics, Recosyn therapy, and odds of analgesic prescription reduction.

Variable	Reduction of at Least 10%		Reduction of at Least 20%	
	Odds Ratio (95% CI)	*p*-Value	Odds Ratio (95% CI)	*p*-Value
Analgesic pills amount three months prior to injection (per 10 pills)	0.98 (0.96–1.00)	0.041	0.97 (0.96–0.99)	0.006
Age < 60 years	Reference		Reference	Reference
Age 60–69 years	1.14 (0.82–1.59)	0.436	1.20 (0.86–1.67)	0.282
Age ≥ 70 years	0.67 (0.55–0.88)	0.005	0.70 (0.53–0.92)	0.012
Women	Reference		Reference	Reference
Men	1.37 (1.07–1.75)	0.014	1.41 (1.10–1.81)	0.007
Statutory health insurance coverage	Reference		Reference	Reference
Private health insurance coverage	0.96 (0.74–1.25)	0.765	0.98 (0.75–1.26)	0.851
Treated by general practitioner	Reference		Reference	Reference
Treated by orthopedist	2.28 (1.71–3.04)	<0.001	2.26 (1.70–3.00)	<0.001
Recosyn	1.64 (1.05–2.58)	0.032	1.51 (0.97–2.34)	0.068
Other drugs	Reference		Reference	Reference

## Data Availability

The data used in this study are subject to strict privacy and confidentiality requirements and therefore cannot be made publicly available. Legal restrictions prohibit the sharing of the raw data with third parties.
